# Engineered Resistance to *Plasmodium falciparum* Development in Transgenic *Anopheles stephensi*

**DOI:** 10.1371/journal.ppat.1002017

**Published:** 2011-04-21

**Authors:** Alison T. Isaacs, Fengwu Li, Nijole Jasinskiene, Xiaoguang Chen, Xavier Nirmala, Osvaldo Marinotti, Joseph M. Vinetz, Anthony A. James

**Affiliations:** 1 Department of Microbiology and Molecular Genetics, School of Medicine, University of California, Irvine, California, United States of America; 2 Division of Infectious Diseases, Department of Medicine, University of California-San Diego School of Medicine, La Jolla, California, United States of America; 3 Department of Molecular Biology and Biochemistry, University of California, Irvine, California, United States of America; 4 Department of Parasitology, School of Public Health and Tropical Medicine, Southern Medical University, Guang Zhou, GD, China; 5 Department of Entomology and Nematology, University of Florida, Gainesville, Florida, United States of America; 6 USDA/ARS, Center for Medical, Agricultural and Veterinary Entomology, Gainesville, Florida, United States of America; University of Notre Dame, United States of America

## Abstract

Transposon-mediated transformation was used to produce *Anopheles stephensi* that express single-chain antibodies (scFvs) designed to target the human malaria parasite, *Plasmodium falciparum*. The scFvs, m1C3, m4B7, and m2A10, are derived from mouse monoclonal antibodies that inhibit either ookinete invasion of the midgut or sporozoite invasion of salivary glands. The scFvs that target the parasite surface, m4B7 and m2A10, were fused to an *Anopheles gambiae* antimicrobial peptide, Cecropin A. Previously-characterized *Anopheles cis*-acting DNA regulatory elements were included in the transgenes to coordinate scFv production with parasite development. Gene amplification and immunoblot analyses showed promoter-specific increases in transgene expression in blood-fed females. Transgenic mosquito lines expressing each of the scFv genes had significantly lower infection levels than controls when challenged with *P. falciparum*.

## Author Summary

Malaria eradication will require vector-control strategies that are both self-sustaining and not affected by migration of infected humans and mosquitoes. Replacement of wild malaria-susceptible mosquito populations with transgenic strains refractory to parasite development could interrupt the cycle of disease transmission and support eradication efforts. Production of *P. falciparum*-resistant mosquitoes is a necessary first step towards investigating the population replacement strategy. Here we show that *An. stephensi* engineered to produce *P. falciparum*-targeting effector molecules are resistant to this important human malaria parasite. Two of the three effector molecules represent a novel combination of components derived from the immune systems of mosquitoes and mice. An important feature of these molecules is that they are unlikely to significantly harm the mosquito, as the mosquito component is an *Anopheles* antimicrobial peptide with activity against *Plasmodium*, while the other component is based on a murine antibody selected for its ability to bind specifically to a parasite protein. Transgenes with this design coupled with a gene-drive system could be used alongside vaccines and drugs to provide sustainable local elimination of malaria as part of a long-term strategy for eradication.

## Introduction

*Plasmodium falciparum*, a causative agent of human malaria, is a vector-borne parasite that is responsible for more than 500 million clinical disease cases each year [1]. The selection of insecticide-resistant mosquitoes and drug-resistant parasites necessitates an ongoing search for new disease-control methods. A proposed strategy for interrupting transmission is to replace wild, malaria-susceptible mosquito populations with transgenic, *Plasmodium*-resistant mosquitoes [2]–[4]. Key components of this approach are effector molecules that inhibit parasite development when expressed from a transgene. The mechanisms by which effector molecules function can vary greatly, as the development of the malaria parasites within mosquitoes involves several transitions of environment, physiology and morphology [5].

When mosquitoes feed on infected humans, they ingest parasites in the form of gametocytes. These produce gametes that fuse to form diploid zygotes that develop into the motile ookinetes. The ookinetes invade and traverse the mosquito midgut epithelium and then rest beneath the basal lamina of the midgut, forming oocysts. Thousands of sporozoites develop within the oocysts before budding out into the circulatory fluid, the hemolymph, and invading the salivary glands. Several effector molecules have been tested for their ability to target the parasite during either early sporogony in the midgut, or late sporogony in the hemolymph or salivary glands [5]–[8]. An effector mechanism based on the mosquito signaling protein Akt is the only one to date shown to inhibit completely *P. falciparum* development in a transgenic *Anopheles* mosquito [7].

One effector molecule strategy exploits the finding that monoclonal antibodies (mAbs) that recognize surface-bound or secreted parasite molecules can inhibit pathogen development [9]–[14]. Two mAbs, 4B7 and 1C3, target parasites early in their development within mosquitoes. 4B7 binds *P. falciparum* surface protein Pfs25, a molecule expressed on the surface of ookinetes, and inhibits parasite development completely when fed to *Anopheles* mosquitoes in a gametocytemic bloodmeal [9]. In contrast, 1C3 binds a parasite-secreted enzyme, *P. falciparum* chitinase 1, and inhibits oocyst formation of *P. falciparum* when incorporated into infectious bloodmeals [10]. A third mAb, 2A10, binds *P. falciparum* circumsporozoite protein (CSP), and when pre-incubated with sporozoites, greatly decreases their ability to infect cultured hepatocytes [11], [12].

Although the size and complexity of mAbs exclude them from consideration as potential effector molecules, single-chain antibodies (scFvs), which retain the binding specificity of a mAb, are much smaller and can be produced from a single transcription unit [15]. An scFv targeting the *P. gallinaceum* CSP inhibited sporozoite invasion of salivary glands in *Aedes aegypti* in both transient assays and transgenic mosquitoes [13], [16]. *Anopheles stephensi* fed *Escherichia coli* expressing an anti-*P. berghei* scFv-immunotoxin were shown to have significantly-reduced oocyst densities when fed on parasite-infected mice [14]. Furthermore, an scFv derived from the 1C3 mAb reduced significantly *P. falciparum* parasite transmission to mosquitoes [17]. The experiments described in the work presented here test the scFv-based strategy on human malaria parasites in transgenic mosquitoes and support the further development and evaluation of these molecules as disease-control tools.

scFvs based on the 1C3, 4B7 and 2A10 mAbs were expressed in transgenic *An. stephensi* and their efficacy tested in parasite challenge assays with *P. falciparum*. *Anopheles stephensi* was chosen because it is a significant vector of urban malaria transmission in the Indian subcontinent and is an efficient model for transgenic research. To distinguish the novel scFvs developed in this study, we refer to them as “modified” 1C3, 4B7 or 2A10 (m1C3, m4B7, m2A10). For the m4B7 and m2A10 transgenes, the *An. gambiae Cecropin A* gene (*AgCecA*) was joined to the scFv gene to form a single open reading frame (ORF). Cecropin A is an antimicrobial peptide that has microbiocidal activity against both gram-negative and gram-positive bacteria, as well as multiple *Plasmodium* species [18], [19]. This broad activity is due to its ability to form large pores in cell membranes [20]. With the addition of cecropin A, the m4B7 and m2A10 scFvs possess both parasite-binding and antimicrobial activity. The cecropin A peptide was not joined to m1C3 as the target of this scFv is a secreted molecule [17].

*Anopheles gambiae carboxypeptidase A* (*AgCPA* [21], [22]) gene regulatory sequences were included in m4B7 and m1C3 transgenes to coordinate their expression with the development of ookinetes. *Anopheles stephensi vitellogenin 1* (*AsVg1* [23]) regulatory elements were joined to the m2A10 scFv to direct transgene expression in the female fat body. Thus, m2A10 secreted from the fat body into the hemolymph could encounter sporozoites migrating to the salivary gland. When challenged in multiple experiments with *P. falciparum* infectious gametocyte cultures, scFv-expressing transgenic lines displayed statistically-significant, reduced mean intensities of infection and in most trials lower parasite prevalence when compared to control mosquitoes.

## Results

### Transgene assembly, transgenesis, and gene copy-number analyses

**Figure 1:**
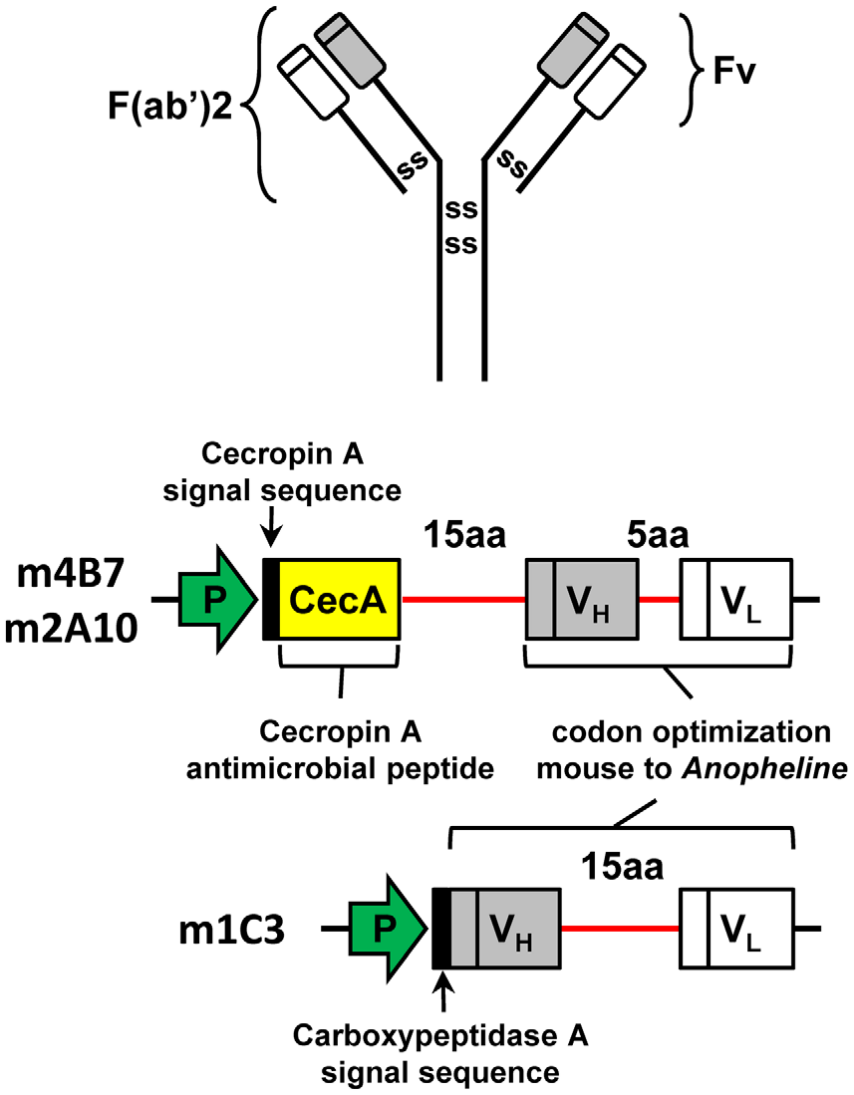
A model of the modified scFv transgenes. A mature mouse immunoglobulin molecule consists of two heavy- and light-chain polypeptides each linked through disulfide (ss) bonds (top image). The single-chain antibodies are composed of the variable regions (Fv) of the heavy (V_H_) and light (V_L_) chains (gray and open boxes, respectively) of a mouse monoclonal antibody. The m4B7 and m2A10 scFv transgenes encode a short polypeptide linker of 5 amino acids (5aa) between V_H_ and V_L_. These transgenes include sequence for a long polypeptide linker of 15 amino acids (15aa) joining the V_H_ to the *An. gambiae* Cecropin A peptide (CecA), including its signal sequence. The V_H_ region present in the m1C3 transgene is joined to the *An. gambiae Carboxypeptidase A* gene signal sequence, and joined by a long polypeptide linker to the V_L_ region. Select codons in the variable region genes were codon-optimized to facilitate efficient translation. *Anopheles* promoter sequences (P) were joined to the scFvs to direct tissue-specific transgene expression.

**Figure 2:**
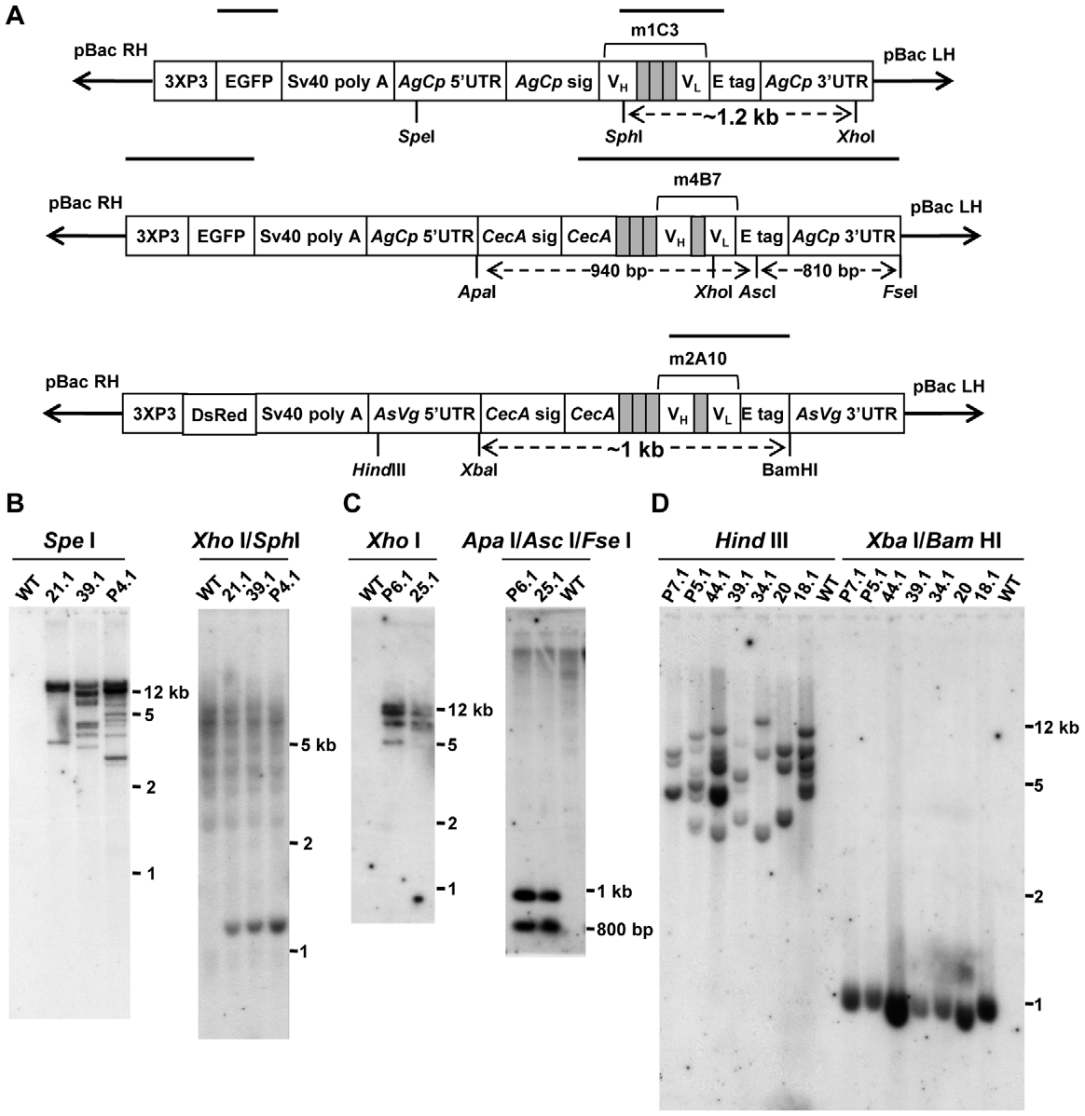
Southern blot analyses of m1C3, m4B7, and m2A10 transgenic lines. (A) Schematic representations of the single-chain antibody (scFv) transformation constructs. The scFv heavy (V_H_) and light (V_L_) variable region genes in the m1C3 construct are joined by sequence encoding a long polypeptide linker (multiple grey boxes). The sequence encoding the V_H_ is joined to the *An. gambiae carboxypeptidase A* signal sequence (*AgCp* sig). In the m4B7 and m2A10 constructs, the *An. gambiae Cecropin A* gene (*CecA*), including signal sequences (*CecA* sig), is joined by sequence encoding a long polypeptide linker to the scFv V_H_ and V_L_ genes. The V_H_ and V_L_ genes are joined by a short polypeptide linker (single grey box). All three scFvs are joined to an epitope tag (E tag). The CecA-m2A10 effector gene is flanked by *An. stephensi vitellogenin 1* regulatory sequences (*AsVg* 5′ UTR, 3′UTR), while the m1C3 and CecA-m4B7 genes are flanked by *AgCP A* regulatory sequences (*AgCp* 5′ UTR, 3′UTR). A transformation marker (EGFP or DsRed) joined to the Pax3 (3xP3) promoter and the SV40 polyadenylation sequence also is contained between the *piggyBac* transposase arms (pBacLH, RH). Select restriction endonuclease sites present in the transgene are indicated in the diagram. Probes used to identify the integrated transgenes are indicated by horizontal bars above each schematic representation. (B) Genomic DNA from m1C3 and wild-type control females was digested and hybridized to either an EGFP probe (left) or an m1C3 probe (right). (C) Genomic DNA from m4B7 and wild-type control females was digested and hybridized to either an EGFP probe (left) or an m4B7/*AgCPA* 3′UTR probe (right). (D) Genomic DNA from m2A10 and wild-type control females was digested and hybridized to an m2A10 probe. The restriction endonucleases used in each experiment are listed above the blot, and the identity of each transgenic line is listed above each lane. The locations of molecular weight markers are indicated in kilobase pairs (kb).

The scFv genes were synthesized commercially to incorporate either the *AgCPA* signal sequence or the entire *AgCecA* ORF (Figure 1). Codons corresponding to the amino acids serine, proline, alanine, threonine, and arginine displayed the greatest frequency bias differences between *Mus musculus* and *An. gambiae* (Table S1) [24], and these were replaced in the mouse-derived scFv sequences by those favored by the mosquito. DNA sequence encoding a short polypeptide linker (five amino acids) was used to join the heavy- and light-chain variable fragments of m4B7 and m2A10 scFvs and a longer linker (encoding 15 amino acids) joined the two corresponding moieties of m1C3. Long linkers permit intramolecular pairing of variable fragments, while short linkers favor the intermolecular joining of scFv molecules to form multimers containing multiple antigen recognition sites [25]. The m1C3 and m4B7 scFv genes were joined to *AgCPA* regulatory elements and inserted into a pBac [3xP3-EGFP] plasmid to construct the transformation vectors (Figure 2). Similarly, the m2A10 scFv gene was joined to *AsVg1* regulatory elements and inserted into a pBac [3xP3-dsRed] plasmid.

The three transformation plasmids pBac [3xP3-EGFP]-m1C3, pBac [3xP3-EGFP]-m4B7 and pBac [3xP3-dsRed]-m2A10 were injected into 980, 615 and 765 embryos, respectively. Three transgenic m1C3 mosquito lines (21.1, 39.1 and P4.1) were established from EGFP-positive families derived from 78 surviving adults. Two transgenic m4B7 mosquito lines (25.1 and P6.1) were established from 89 adults, and seven transgenic m2A10 mosquito lines (18.1, 20, 34.1, 39.1, 44.1, P5.1 and P7.1) were established from 105 adults.

Southern blot analyses were used to verify transgene insertions and to determine the number of integrated constructs in each line (Figure 2). Hybridization of an m1C3 probe to genomic DNA digested with both *Sph*I and *Xho*I restriction endonucleases produced a diagnostic fragment of ∼1.2 kilobase pairs (kb) in transgenic samples, confirming m1C3 integration. Genomic DNA digested with *Spe*I and hybridized to an EGFP probe produced multiple fragments in each transgenic sample, indicating that there were at least three, nine, and ten copies in lines 21.1, 39.1, and P4.1, respectively. Genomic DNA digested with *ApaI*, *Asc*I, and *Fse*I, and hybridized to a probe complementary to the m4B7 gene and the *AgCPA* 3′UTR produced two diagnostic fragments of 940 and 810 base pairs (bp), verifying transgene insertion. A second blot, comprising *Xho*I-digested genomic DNA recovered from transgenic mosquitoes and hybridized with a 3XP3 EGFP probe, revealed several fragments in each sample, indicating that at least four copies of the m4B7 transgene were present in each line. Lastly, genomic DNA digested with both *Xba*I and *Bam*HI and hybridized to an m2A10 probe produced an ∼1 kb diagnostic fragment in each transgenic sample. The same probe hybridized to *Hind*III-digested genomic DNA bound multiple DNA fragments in each m2A10 sample, indicating the presence of six, three, three, four, six, seven and three copies in transgenic lines 18.1, 20, 34.1, 39.1, 44.1, P5.1, P7.1, respectively. Transgenic lines were maintained by intercrossing at each generation. However, selection pressures on individual transgene insertions, small founding colony sizes and independent assortment likely result in loss over time of some of the insertions.

### Characterization of transgene expression

Reverse-transcriptase-PCR (RT-PCR) and Real-time quantitative RT-PCR (RT-qPCR) were used to evaluate the presence and relative abundance of m1C3, m4B7 or m2A10 transcription products in non-blood-fed and blood-fed mosquitoes in all of the established transgenic lines. No significant correlation was seen between transgene copy number and amount of transcription product detected (data not shown). Therefore, the lines m1C3 P4.1, m4B7 25.1 and m2A10 44.1, each of which displayed the highest levels of transcript accumulation in their respective group, were selected for use in all further analyses. Southern blot analyses of the generations of m1C3 P4.1, m4B7 25.1 and m2A10 44.1 used in the challenge assays indicated the presence of eight, four, and four copies of the respective transgenes.

**Figure 3:**
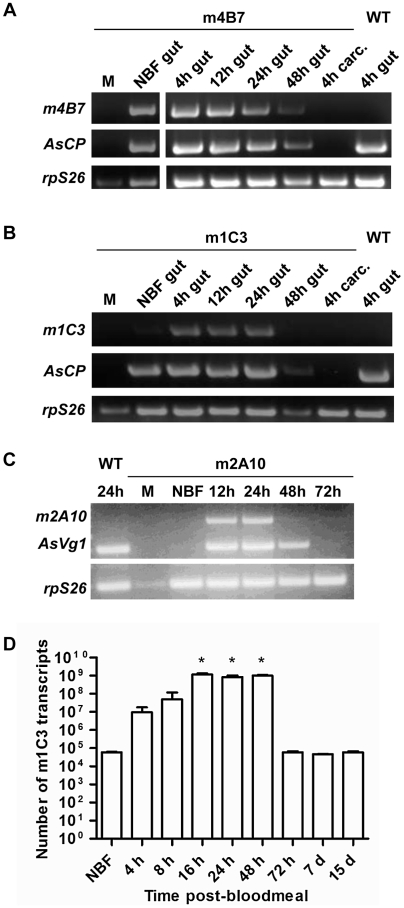
Expression of m1C3 P4.1, m4B7 25.1, and m2A10 44.1 transgenes. Representative results of gene amplification experiments show tissue- (A, B, C), sex- (A, B, C) and stage-specific (C) accumulation of transgene transcription products. (A) RT-PCR was used to detect m4B7 and endogenous *An. stephensi* carboxypeptidase A (*AsCPA*) transcript in RNA isolated from either whole m4B7 25.1 transgenic males (M) or dissected wild-type (WT) control, non-blood-fed m4B7 25.1 transgenic females (NBF) and m4B7 25.1 transgenic females at multiple post-bloodmeal time points (indicated above each lane). For female samples, RNA was extracted from either gut or carcass homogenates. (B) RT-PCR was used to detect m1C3 and endogenous *An. stephensi* carboxypeptidase A (*AsCPA*) transcript as described in (A). The *An. stephensi* S26 ribosomal protein transcript (*rpS26*) was amplified as a loading control in all RT-PCR experiments. (C) Accumulation of m2A10 44.1 and endogenous *An. stephensi* vitellogenin (*AsVg1*) transcript in transgenic males, non-blood-fed females, and females at multiple post-bloodmeal time points was assayed by RT-PCR. RNA was isolated from adult whole-body homogenates of wild-type control and m2A10 44.1 transgenic mosquitoes. RNA from wild-type control females was included as both a positive control for *AsVg1* expression and as a negative control for m2A10 44.1 amplification. Parallel reactions in which the reverse transcription reaction step were omitted demonstrated that the observed amplification products originated exclusively from RNA (data not shown). (D) Developmental expression of m1C3 mRNA. m1C3 mRNA abundance in the midguts of females is described as transcript numbers in log-scale. Each data point represents the average of three independent biological replicates. An asterisk indicates a significantly larger number of transcripts when compared to NBF (paired T-test, one tailed p-value, 16 h p = 0.0050, 24 h p = 0.0067, 48 h p = 0.0018).

Transgene-specific transcript accumulation profiles detected by RT-PCR were similar in mRNA samples prepared from the dissected midguts of m1C3 P4.1 and m4B7 25.1 females (Figure 3). Both lines showed constitutive accumulation in midguts from non-blood fed mosquitoes. In addition, each line showed accumulation of their respective mRNAs at 4 hours post-bloodmeal (hPBM), and signals were evident at 12 and 24 hPBM. m4B7 transcript also could be detected at low levels at 48 hPBM. No amplification products were produced from mRNA prepared from female carcasses or males of each line. As expected, control reactions using mRNA from midguts dissected at 4 hPBM from wild-type, non-transgenic females were negative. RT-qPCR analysis at multiple post-bloodmeal time points was used as an independent measure of m1C3 P4.1 expression. The highest measured level of m1C3 mRNA, 10,000-fold above the control level, was observed in the 16 hPBM midgut sample (paired T-test, one tailed p-value = 0.005), but similar elevated levels also were seen at 24 and 48 hPBM. At this time, we cannot account for the difference in the RT-PCR and RT-qPCR results at 48 hPBM, although this could result from individual females that responded differentially to the feeding regimen. This difference is not expected to have affected the outcome of the challenge experiments because this scFv targets parasites within the first 24 hPBM.

Immunoblot analyses of m1C3 and m4B7 transgenic mosquitoes were unproductive despite repeated attempts. Although high levels of proteinase inhibitors were used during sample preparation, it is possible that the transgene products were degraded quickly in the strong digestive milieu of the post-feeding midgut lumen.

Transgene transcripts detected in whole transgenic m2A10 44.1 females showed sex- and stage-specificity (Figure 3). No signals were seen in samples derived from mRNA prepared from males and non-blood fed transgenic or control wild-type, non-transgenic females. Specific transcript accumulation was evident in m2A10 44.1 females at 12 and 24 hPBM in an expression pattern similar to that of endogenous *AsVg1*, but was not as abundant at 48 hPBM.

**Figure 4:**
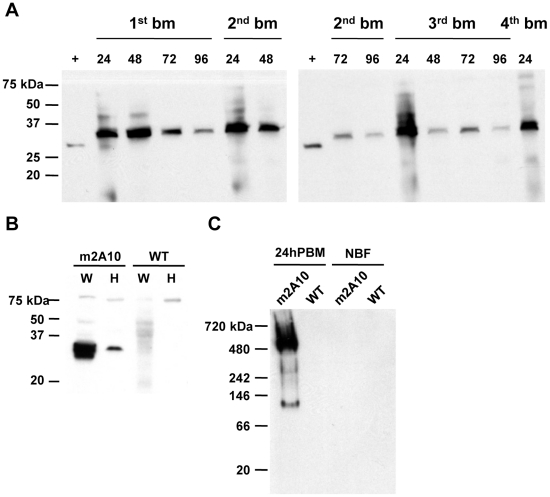
Bloodmeal-induced expression of m2A10 scFv. (A) Immunoblot analyses of transgenic m2A10 44.1 *An. stephensi*. An anti-E tag antibody was used to detect m2A10 protein in whole body homogenates of transgenic females. Purified m2A10 scFv protein, which contained an E tag but did not contain Cecropin A, was used as a positive control (+). Female mosquitoes were given bloodmeals (bm) once every five days, and examined at 24, 48, 72, and 96 hours after each meal. (B) An immunoblot of both whole females (W) and a hemolymph preparation of females (H) from transgenic m2A10 44.1 and wild-type control mosquitoes (WT) 48 hours post-bloodmeal. Anti-E tag antibody was used for detection of m2A10. (C) An immunoblot of m2A10 and wild-type control female hemolymph samples, prepared in non-denaturing conditions (left panel) and probed with anti-E tag antibody. Non-bloodfed (NBF) and 24 hours post-bloodmeal (24hPBM) females were compared. NativeMark protein standard (Invitrogen) was used for molecular weight estimation. Control images of the Coomassie-stained gels are provided in Figure S1.

While expression of the m1C3 and m4B7 scFvs is necessary only during the first 24 hours post-bloodmeal, expression of m2A10 must be sustained over several days, as oocysts can mature asynchronously [26]. Denaturing immunoblot analyses were performed on m2A10 44.1 females sampled over the course of four bloodmeals to evaluate whether protein expression could be induced repeatedly (Figure 4; Figure S1). Anti-E tag antibody specifically detected a polypeptide with an approximate M_r_ of 32 kiloDaltons (kDa), consistent with the predicted size of m2A10 protein, in transgenic blood-fed females at 24, 48, 72 and 96 hPBM. The continuous presence of m2A10 was detected in females that were given bloodmeals once every five days. Expression of m2A10 also was observed at 12 hPBM in additional immunoblot analyses (data not shown). Immunoblots of hemolymph samples indicated that m2A10 protein was present in the hemolymph of blood-fed transgenic females (Figure 4). Immunoblot analyses of hemolymph samples analyzed in non-denaturing conditions detected m2A10 protein in several multimeric conformations with estimated M_r_s of 125, 223, 284, and 485 kDa.

### *Plasmodium falciparum* challenge of transgenic and control mosquitoes

Parasite challenge experiments were performed to test the efficacy of the anti-pathogen effector molecules. Transgenic and control mosquitoes ingested blood containing *P. falciparum* gametocytes through a membrane-feeding apparatus. Control mosquitoes for most experiments were non-transgenic (wild-type) mosquitoes. In addition, the oocyst prevalence and mean intensities of infection of a group of m2A10 44.1 females were examined for each challenge experiment to determine whether transgenesis alone had an impact on parasite development.

**Figure 5:**
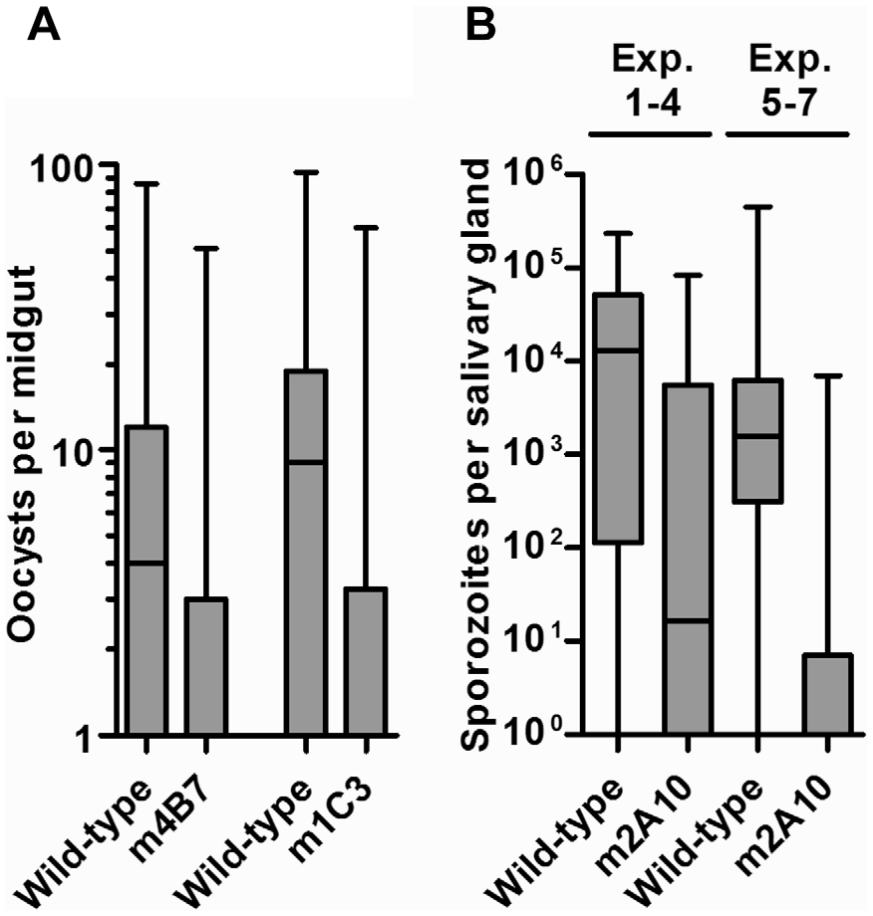
*P. falciparum* oocyst or sporozoite burden in transgenic and wild-type control mosquitoes. Box plots display the observed distribution of oocyst per midgut or sporozoite per salivary gland pair values for wild-type control mosquitoes and m1C3 P4.1, m4B7 25.1, or m2A10 44.1 transgenic mosquitoes. The boxes in each panel represent from bottom to top the 25^th^ to 75^th^ percentiles, while the vertical lines and small horizontal bars delimit the minimum and maximum values. Horizontal bars within the boxes indicate the median value of each group. (A) Plots of data from all challenge experiments with m4B7 25.1 and m1C3 P4.1 mosquitoes. (B) The plot represents data from m2A10 44.1 experiments 1–4 and 5–7 separately, as indicated.

The effect of transgene expression on parasite development for both the m4B7 25.1 and m1C3 P4.1 transgenic lines was measured by comparing the number of oocysts in transgenic and control mosquito midguts at nine days after the infectious bloodmeal (Table 1; Figure 5). The mean intensities of oocyst infection were reduced by 37–81% in three challenge experiments (1, 2 and 3, Table 1) of m4B7 25.1. However, the mean intensities of infection were reduced by only 29–36% in two experiments (4 and 5, Table 1) in which control mosquitoes had greater than 17 oocysts per midgut. Mean intensities of oocyst infection were reduced by 47–73% in mosquitoes expressing m1C3 when compared to controls. Furthermore, with the exception of the high infection-level experiments (4 and 5, Table 1), both m1C3 P4.1 and m4B7 25.1 transgenic mosquitoes had lower prevalence of infections than controls.

**Table 1:** Oocyst prevalence and mean intensity of infection in *P. falciparum*-infected mosquitoes.

Exp.	Strain	Oocyst prevalence^a^ (n)	Mean intensity of oocyst infection^a^ +/− SD (n)	P^b^
1	Control	84%(45)	15.3+/−12.3 (38)	<0.0001
	m4B7	48% (50)	2.9+/−2.4 (24)	
2	Control	70% (67)	4.6+/−4.4 (47)	<0.0001
	m4B7	41% (93)	2.9+/−2.9 (38)	
3	Control	77% (64)	6.3+/−4.5 (49)	<0.0001
	m4B7	40% (92)	2.4+/−1.9 (37)	
4	Control	88% (48)	19.8+/−21.4 (42)	0.3736
	m4B7	88% (43)	14.1+/−10.5 (38)	
5	Control	83% (46)	17.4+/−17.8 (38)	0.2883
	m4B7	88% (41)	11.2+/−9.5 (36)	
6	Control	83% (23)	22.5+/−13.5 (19)	0.0068
	m1C3	64% (22)	12.0+/−11.6 (14)	
7	Control	86% (22)	38.8+/−36.8 (19)	0.0007
	m1C3	46% (24)	14.3+/−18.1 (11)	
8	Control	48% (23)	7.1+/−5.8 (11)	0.0004
	m1C3	9% (33)	3.0+/−2.0 (3)	
9	Control	67% (12)	10.5+/−4.4 (8)	0.0043
	m1C3	39% (23)	3.9+/−3.6 (9)	
10	Control	46% (24)	3.7+/−2.1 (11)	0.0065
	m1C3	15% (20)	1.0+/−0.0 (3)	

a Prevalence reflects the percent of mosquitoes infected; mean intensity of infection reflects the mean number of parasites found in infected mosquitoes.

b A Mann-Whitney *U* test was used to evaluate statistical significance of oocyst mean intensity infection data. A one-tailed P value is listed for each experiment.

Parasite challenge assays of m2A10 44.1 involved dissecting 7–11 mosquitoes of each group 10 days after the infectious bloodmeal to count midgut oocysts and to confirm that both transgenic and control mosquitoes were infected successfully (Table 2). No statistically significant difference in the number of oocysts between transgenic m2A10 44.1 and control mosquitoes was observed (Mann-Whitney *U* test, one-tailed P value, 0.24<p<0.48). The remaining mosquitoes (n = 8–50) in each group were examined 17–19 days after infection for the presence of sporozoites in the salivary glands (Table 2; Figure 5). All mosquitoes were provided an uninfected bloodmeal every five days to maintain expression of m2A10. Engorged and un-engorged females were not separated after the uninfected bloodmeals in experiments 1, 2, 3 and 4, and a 52–84% reduction in mean intensity of sporozoite infection was observed in transgenic mosquitoes when compared to the controls. To obtain a more precise measurement of the effect of m2A10 expression upon *P. falciparum* development, an additional three experiments (5, 6 and 7) were performed in which un-engorged females were discarded after each uninfected bloodmeal. A 96–97% reduction of mean intensity of infection was observed in m2A10 44.1 mosquitoes that fed every five days. Furthermore, m2A10 44.1 mosquitoes in experiment 7 had a 14% prevalence of infection compared to 78% observed in the corresponding control.

**Table 2:** Oocyst and sporozoite prevalence and mean intensity of infection in *P. falciparum*-infected mosquitoes.

Exp.	Strain	Oocyst prevalence^a^ (n)	Mean intensity of oocyst infection^a^ +/− SD (n)	sporozoite prevalence (n)	Mean intensity of sporozoite infection +/− SD (n)	P^b^
1	Control	83% (12)	8.4+/−8.1 (10)	89% (9)	5887+/−5627 (8)	0.0441
	m2A10	82% (11)	5.1+/−4.4 (9)	50% (8)	2810+/−2823 (4)	
2	Control	92% (12)	12.9+/−10.7 (11)	81% (21)	10979+/−15002 (17)	0.0525
	m2A10	92% (12)	10.6+/−8.5 (11)	71% (21)	3521+/−4270 (15)	
3	Control	100% (10)	31.2+/−18.4 (10)	100% (10)	57116+/−62539 (10)	0.0446
	m2A10	90% (10)	34.9+/−29.2 (9)	90% (10)	19173+/−27834 (9)	
4	Control	90% (10)	30+/−19.8 (9)	100% (23)	58199+/−57645 (23)	<0.0001
	m2A10	100% (11)	29.5+/−16.4 (11)	74% (23)	9434+/−17141 (17)	
5	Control	70% (10)	14.6+/−13.9 (7)	98% (49)	19529+/−49509 (48)	<0.0001
	m2A10	70% (10)	15.0+/−12.5 (7)	67% (49)	528+/−1123 (33)	
6	Control	90% (10)	10.2+/−8.0 (9)	98% (50)	17383+/−65246 (49)	<0.0001
	m2A10	90% (10)	8.4+/−6.1 (9)	44% (50)	622+/−1678 (22)	
7	Control	82% (11)	4.7+/−3.2 (9)	78% (50)	17913+/−39420 (39)	<0.0001
	m2A10	80% (10)	4.4+/−3.3 (8)	14% (50)	450+/−1024 (7)	

a Prevalence and mean intensity of infection are as described in Table 1.

b A Mann-Whitney *U* test was used to evaluate statistical significance of sporozoite mean intensity of infection data. A one-tailed P value is listed for each experiment.

## Discussion

Previous evaluations of mosquitoes engineered genetically to express anti-*Plasmodium* effector genes featured analyses of transgene copy numbers, transgene transcription levels, detection of transgene effector proteins, binding of effector molecules to the target parasite stage and a phenotype of reduced parasite mean intensities of infection and prevalence [7], [8], [16], [22], [27]–[29]. Remarkably, no single study includes all of these data and the emphasis has been on the impact of transgene presence on parasite numbers. Expression of the two midgut-directed scFvs, m1C3 and m4B7, was detected by RT-PCR, but not by immunoblots. The rapid degradation of these scFvs in the midgut environment may have inhibited immunoblot detection. However, the observation that m4B7 25.1 and m1C3 P4.1 transgenic mosquitoes have reduced parasite loads supports the conclusion that these scFvs are expressed in the midgut. Both transgene transcription and translation products were detected in m2A10 44.1 mosquitoes. The finding that the immunoblot analyses of non-denatured m2A10 44.1 samples detected the presence of scFv multimers is consistent with the expectation that the short polypeptide linker joining the V_H_ and V_L_ regions promotes intermolecular scFv interactions. The size of these multimers was similar to the predicted sizes of m2A10 multimers comprising four, seven, nine, and fifteen scFv molecules. Such scFv multimers are reported to have high affinity to target epitopes [25].

Both m1C3 and m4B7 expressed in transgenic lines P4.1 and 25.1, respectively, inhibited parasite development during early sporogony, resulting in significantly reduced mean intensities of oocyst infection in eight of ten challenge experiments. The results of two of the m4B7 25.1 challenge experiments are consistent with the interpretation that there is a threshold level of initial parasite density above which this scFv, at the levels expressed in these transgenic lines, cannot efficiently inhibit ookinete development. The finding that m2A10 44.1 and wild-type control mosquitoes did not differ in midgut infection supports the conclusion that transgene integration alone does not necessarily impair parasite development.

When expression of m2A10 in line 44.1 was induced repeatedly by blood feeding, a highly significant decrease in sporozoite load was observed in transgenic mosquito salivary glands. For this transgenic line, the greatest reduction in prevalence was found in an experiment in which the mean intensity of oocyst infection was low. It is likely that these scFvs would effectively impair *P. falciparum* transmission in field conditions, as infected wild-caught *An. gambiae* carry few oocysts. Studies of *An. gambiae* by Billingsley *et al.* [30] and Taylor [31] found mean numbers of oocysts per infected mosquito of 1.55 and 3.38, respectively.

Incorporation of multiple transgenes is typical for *piggyBac*-mediated insertions into *An. stephensi* [22], [23], [32]. Although it is reasonable to expect that higher transgene copy numbers should yield higher expression levels, no statistically-significant correlations have been reported. We hypothesize that many of the multiple copies have little or no expression as a result of position effects, and that the majority of transgene expression comes from single or small numbers of the transgenes. To mitigate copy-number issues, we have used *piggy-Bac*-mediated transposition to integrate target sites for *ϕC31* site-specific recombination into multiple locations in the *An. stephensi* genome and are now testing individual lines for permissiveness for optimum transgene expression [33]. These lines have the added benefit of having been evaluated for the impact on fitness of the introduced exogenous DNA at the specific insertion site, and therefore the effects of anti-pathogen transgene product expression can be measured directly. Furthermore, we are eager to evaluate the phenotype of dual transgenes, for example, those combining m4B7 and 2A10 or m1C3 and m2A10, on parasite mean intensities of infection and prevalence. Additional studies facilitated by this approach could include testing alternate gene regulatory sequences, such as those of the salivary gland-specific anopheline antiplatelet protein or the *An. gambiae* adult peritrophic matrix protein 1, to measure the effect of different transgene expression patterns upon parasite development [34], [35].

Although the scFvs in lines m1C3 P4.1, m4B725.1, and m2A10 44.1 inhibited parasite development significantly, no transgenic line displayed zero prevalence of infection. It has been demonstrated in an avian malaria model system comprising the vertebrate host, *Gallus gallus*, the mosquito host, *Aedes aegypti*, and the parasite, *P. gallinaceum*, that mosquitoes containing as few as 20 sporozoites in their salivary glands infected chickens during a blood meal [16]. This finding supports the conclusion that a target of zero prevalence is necessary for a transgenic mosquito to be incapable of disease transmission in this system. These results are in contrast to reports of experiments with transgenic mosquitoes and a rodent malaria parasite, *P. berghei*, in which the effector molecules SM1, PLA2 and CEL-III show a significant inhibition of parasite development (81.6%, 87% and 84.8%, respectively) [22], [27], [28]. Reductions of mean intensities of *P. berghei* sporozoite infection in salivary glands below ∼400 were sufficient to block transmission. In contrast, experimental infections of humans with *P. vivax* showed that 10 sporozoites were sufficient to cause malaria [36]. We have opted to take the conservative approach and are attempting to achieve zero prevalence of human parasites in mosquito salivary glands [16].

Two anti-*Plasmodium* effector molecule strategies have yielded transgenic mosquitoes with zero prevalence: expression of the signaling molecule Akt, and expression of a combination of Cecropin A and Defensin A [7], [29]. The latter study was conducted with the *P. gallinaceum*/*Ae. aegypti*/*G. gallus* model system. The study of Akt demonstrated the feasibility of producing an *Anopheles* mosquito that is completely resistant to *P. falciparum*, however this effector molecule may not be an optimal component of a population replacement strategy as these mosquitoes have a significantly reduced lifespan [7]. A synthetic peptide designed to interact with *P. yoelii* reduced midgut infections of this parasite by 67–87% in *An. gambiae* but was considerably less efficacious against *P. falciparum* [8]. Quantitative comparisons of the efficacy of alternative effector molecules are hindered currently by differences in expression that result from variations in transgene location and copy number. Site-specific recombination approaches will allow such evaluations in well-characterized ‘docking-site’ mosquito strains [8], [33].

The finding that m2A10 44.1 mosquitoes display up to 97% decreases in the mean intensity of *P. falciparum* infection, as well as decreased prevalence of infection, supports the argument that this scFv may be an effective component of a malaria resistance transgene. The m1C3 and m4B7 scFv genes conferred significant reductions in mean intensities of infection, and if expressed in higher quantities, also may be used in the design of a transgenic, parasite-resistant mosquito. Furthermore, expressing the scFv transgenes in additional malaria vectors, in particular, *An. gambiae*, and challenging these with a variety of *P. falciparum* isolates would help evaluate whether these effector molecules could be used in multiple transmission areas.

The discovery and characterization of several effector molecules that completely inhibit *P. falciparum* development will support the engineering of mosquitoes that express multiple effector molecules. Such mosquitoes may have a reduced likelihood of selecting for resistant parasites. Along with vaccines, drugs, and insecticide-treated nets, parasite-resistant transgenic mosquitoes would be a useful component in a malaria-control strategy, especially in regions where existing interventions have been unable to eliminate disease transmission.

## Materials and Methods

### Mosquito rearing and maintenance

A colony of *Anopheles stephensi* (gift of M. Jacobs-Lorena, Johns Hopkins University) bred in our insectary for >5 years was used in the experiments. The mosquitoes were maintained in conditions that maximize larval nutrition, and adult size and fitness [37]. These conditions include maintenance of cultures at 27°C with 77% humidity and 12 hr day/night, 30 min dusk/dawn lighting cycle. Larvae were fed a diet of powdered fish food (Tetramin) mixed with yeast. Adults were provided water and raisins *ad libitum*. Anesthetized chickens, mice, or rabbits were used for blood feeding. Transgenic and wild-type control mosquitoes used in parasite challenge experiments were reared in parallel using standardized insectary procedures.

### Ethics statement

This study was carried out in strict accordance with the recommendations in the Guide for the Care and Use of Laboratory Animals of the National Institutes of Health. The protocol was approved by the Intuitional Animal Care and Use Committee of the University of California, Irvine (NIH Animal Welfare Assurance number: A3416.01 (approved February 20, 2008), Protocol Number: 1998- 1411 (approved May 21, 2010). The vertebrates used as bloodmeal donors for mosquitoes were anesthetized on a regimen that avoids the build-up of drug tolerance, and all efforts were made to minimize suffering.

### scFv sequence modifications

The sequences of the 4B7 and 2A10 variable heavy- and light-chain regions (V_H_ and V_L_, respectively) were derived from cDNA synthesized from 2A10 and 4B7 hybridoma cell lines (obtained from E. Nardin [New York University], and the Malaria Research and Reference Reagent Resource Center, respectively). 2A10 cDNA was synthesized from total RNA isolated from the hybridoma cell line using primers designed from the known V_H_ and V_L_ sequence [38]. V_H_ and V_L_ cDNA from the 4B7 hybridoma cell line was amplified from its total RNA using the heavy and light primer mixes respectively, provided in the Mouse ScFv Module/Recombinant Phage Antibody System (Amersham Biosciences). The modified scFv genes, including either *AgCPA* signal sequence or the entire *AgCecA* ORF, were synthesized commercially (Epoch Biolabs) to allow for incorporation of novel features. The variable regions of 4B7, 2A10, and 1C3 [17] were optimized by replacing the codons corresponding to the amino acids serine, proline, alanine, threonine, and arginine in the mouse-derived sequences with those favored in *An. gambiae* (Table S1) [24]. For the m4B7 and m2A10 scFvs, the variable regions were joined by sequence encoding a short polypeptide linker, G_4_S. The V_H_ region of the m2A10 and m4B7 scFv sequences were joined to the AgCecA protein-coding sequence by a long polypeptide linker, (G_4_S)_3_ [39]. The variable regions of m1C3 were joined by the same long polypeptide linker. The m2A10 V_L_ was joined to sequence encoding a complete E tag, while the V_L_ of m4B7 and m1C3 were joined to sequence encoding a partial E tag. For m4B7 and m1C3, the remaining E tag coding sequence was joined to the partial E tag at a later cloning step.

### Transformation plasmid assembly

The pBacDsRed-AsVg5′-m2A10′-AsVg3′ plasmid was produced in two cloning steps. First, m2A10 sequence from the commercially-synthesized pBSKm2A10 plasmid replaced the CFP gene of pSLfa-AsVg5′-CFP-AsVg3′ [23] using *Xba*I and *Bam*HI sites. Second, the AsVg5′-m2A10-AsVg3′ sequence was joined to pBacDsRed [37] using *Asc*I sites. A pSLfa-AgCP5′-4B7-AgCP3′ plasmid supplied the AgCP regulatory sequences, as well as a partial E tag sequence, for both the pBacEGFP AgCP5′-m1C3-AgCP3′ and the pBacEGFP AgCP5′-m4B7-AgCP3′ plasmids. The pSLfa-AgCP5′-4B7-AgCP3′ plasmid was cloned in several steps. First, the Mouse ScFv Module/Recombinant Phage Antibody System (Amersham Biosciences) was used to produce a single chain antibody from V_H_ and V_L_ cDNA from the 4B7 cell line. The scFv was cloned into the pCANTAB 5E vector in frame with the E-Tag at the C terminus. *Bcl*I sites were added to both ends of 4B7 scFv by amplification using primers 4B7BclF [5′-CGTGATCAGTGAAGCTGGTGGAGTCT-3′] and 4B7BclR [5′-CGTGATCACTATGCGGCACGCGGTT-3′] from the 5′ and 3′ end and cloning into pCR4Blunt-TOPO. The pGEMT[AgCP-SM1] plasmid, containing AgCP (AGAP009593) regulatory regions, was generously provided by Dr. Marcelo Jacobs-Lorena [22]. The *Bcl*I-cut 4B7 scFv fragment from TOPO [4B7*Bcl*I] was sub-cloned into the *Bam* HI sites of pGEMT[AgCP-SM1] thereby swapping the SM1 fragment with 4B7. AgCP5′-4B7-AgCP3′ was cloned subsequently into pSLfa1180fa [40] using enzymes *Sac*II and *Sal*I. *Apa*I and *Sgr*AI were used to replace the 4B7 region of pSLFA-AgCP5′-4B7-AgCP3′ with the commercially-synthesized m4B7 gene. AgCP5′-m4B7-AgCP3′ sequence was then joined to pBacEGFP [40] using a 5′ blunt ligation of *Asc*I and *Kpn*I sites, and a 3′ *Fse*I ligation. To assemble the m1C3 transformation plasmid, the enzymes *Apa*I and *Bam*HI were used to replace the 4B7 region of pSLfa-AgCP5′-4B7-AgCP3′. AgCP5′-m1C3-AgCP3′ was then joined to pBacEGFP using *Asc*I restriction sites.

### Microinjection and southern hybridization analyses

Microinjection of the pBac [3xP3-EGFP]-m1C3, pBac [3xP3-EGFP]-m4B7 or pBac [3xP3-dsRed]-m2A10 plasmids with the *piggyBac* helper plasmid was performed as described previously, except that 0.1 mM p- nitrophenyl p′-guanidinobenzoate was omitted from isotonic buffer [41]. Each G_0_ male was mated with 15 virgin females and groups of 5–10 G_0_ females were mated with 5 males, and G_1_ progeny were screened as larvae with UV-fluorescence microscopy for the presence of the marker genes. Standard Southern blotting and hybridization techniques were used to detect transgene integration [42]. Genomic DNA was extracted from groups of six transgenic or wild-type control females as described previously, except that DNA pellets were re-suspended in 100 µl of dH_2_O [43]. The probe used to identify m1C3 integration was amplified from a plasmid thought to contain the EGFP ORF, but which in fact contained the ECFP ORF. These two ORFs share 99% nucleotide sequence identity, so it is likely that the ability of the probe to hybridize to the integrated gene was affected negligibly. The EGFP probe was generated from pMos[3xP3-EGFP] [37] using *Xba*I and *Sac*I enzymes. The m4B7 probe was generated through a restriction digest of the pBac [3xP3-EGFP]-m4B7 plasmid with both *Nae*I and *Fse*I. The m2A10 probe was generated through a restriction digest of the pBSK-m2A10 plasmid with both *Bam*HI and *Bst*BI. Probes were labeled with ^32^P using the Megaprime DNA labeling system (Amersham).

### RT-PCR

Total RNA was isolated from whole or dissected mosquitoes using Trizol (Invitrogen). For m2A10 RT-PCR analyses, 10 males or 2–3 whole females were used for each RNA preparation. One microgram of RNA was treated with DNAseI (Promega) for each 50 µl RT-PCR reaction. For m4B7 and m1C3 RT-PCR analyses, 6 males, 4–15 female midguts, or 4 female carcasses were used for each RNA preparation. Two hundred fifty nanograms of RNA were treated with DNAseI for each 12.5 µl RT-PCR reaction. Gene-specific primers and a OneStep RT-PCR Kit (Qiagen) were used for amplification of diagnostic products from *m2A10*, *m4B7*, *m1C3*, *AsCPA* [32], *AsVg1* [23], or *An. stephensi* ribosomal protein S26 gene [23] transcripts (Table S2). For m2A10 RT-PCR analyses, amplification of diagnostic products from AsVg1 and ribosomal protein S26 gene-specific primers was performed in a single reaction. Diagnostic amplification reactions for the m4B7 25.1 and m1C3 P4.1 lines were initiated with one cycle at 50°C for 30 m, one cycle at 95°C for 15 m, 32 cycles denaturation at 94°C for 30 s, annealing at a reaction-specific temperature (Table S2) for 30 s, and extension at 72°C for 1 m, followed by a final extension at 72°C for 10 m. Diagnostic amplification reactions for the m2A10 44.1 line were performed as described, except that 30 cycles of amplification were completed. For each sample, an additional control RT-PCR reaction tested for the presence of genomic DNA contamination using ribosomal protein S26 gene primers but omitting the reverse transcription step. Multiple biological replicates (≥2) were performed for selected time points for each of the RT-PCR series of experiments.

### Real-time quantitative RT-PCR analysis

Female mosquito midguts and carcasses and male midguts were dissected in phosphate-buffered saline (PBS), homogenized in Trizol Reagent (Invitrogen), and total RNA extracted. Midguts were dissected at different time points (4 h, 8 h, 16 h, 24 h, 48 h, 72 h, 7 d, and 15 d) after a bloodmeal. RNA was treated with DNase I (Invitrogen) at 1 U/µg RNA to remove potential genomic DNA contamination. Further purification was performed using a DNA-free kit (Ambion). A total of 0.4 µg of RNA was used for reverse transcription in a reaction volume of 20 µl using ThermoScript RT-PCR System (Invitrogen). Real-time quantitative PCR was performed on an Opticon 2 Real-Time PCR Detection System using the Opticon Monitor software version 3.1 (Bio-Rad laboratories). m1C3 expression was quantified with Platinum SYBR Green qPCR SuperMix UDG with ROX (Invitrogen) using gene-specific primers (Table S2) to amplify a diagnostic fragment 211 bp in length. A series of quantitative standards were generated from serial 10-fold dilutions (a range of 10^10^-1 molecules) of TOPO-m1C3scFv, in which full-length m1C3 was cloned. Each assay was run in triplicate wells in a 25 µl final reaction volume containing 2.5 µl of Platinum SYBR Green, 400 nM each forward and reverse primer, and 2.5 µl cDNA sample. Each run included negative controls consisting of wild-type control cDNA and water instead of cDNA. The amplification protocol consisted of 2 min at 50°C, 2 min at 95°C, followed by 40 cycles of amplification (94°C for 15 s, 60°C for 45 s, plate read of SYBR Green I fluorescence), after which a melting-curve reaction was conducted from 42°C to 95°C with plate readings every 1°C. GraphPad Prism software was used to calculate statistical significance using paired T-tests.

### Immunoblot analysis

Mosquitoes were blood-fed on chickens and homogenized in a protease inhibitor solution made from complete mini (Roche) and Pefabloc SC (Roche). An equal volume of Laemmli sample buffer (Bio-Rad) with 0.1 M dithiothreitol was added. Homogenates were separated on a 12% Tris-HCl polyacrylamide gel in 1×Tris/Glycine/SDS buffer (Bio-Rad), transferred to Immun-Blot PVDF membrane (Bio-Rad), and incubated with goat anti-E tag polyclonal antibody conjugated to horse radish peroxidase (Abcam). ECL Plus Western Blotting Detection Reagents (GE Healthcare) were used to detect bound antibody. Ten females were used for each hemolymph sample preparation. Legs were removed with forceps and the proboscis was cut with a scissor. Individuals were inserted into a pipette tip plugged with glass wool and threaded through a 0.5 ml tube placed in a 1.5 ml collection tube. Centrifugation at 530 g for 10 min at 4°C extracted hemolymph. Each hemolymph sample was mixed with 25 µl 0.15 M NaCl and centrifuged at 2040 g for 5 min at 4°C. Fifteen microliters of the middle fraction of the sample was transferred to a new 1.5 ml tube, to which 10 µl of the protease inhibitor solution was added. For immunoblots with non-denatured samples, native sample buffer (Bio-Rad), 4–15% Tris-HCl polyacrylamide gels (Bio-Rad), 1×Tris/Glycine electrophoresis buffer (Bio-Rad), and native transfer buffer (25 mM Tris, 25 mM Glycine, pH 9.2) were used.

### Parasite challenge experiments

Four to six day-old transgenic and wild type female mosquitoes were fed with *P. falciparum* NF 54 gametocytes using a membrane feeding apparatus. After 15 min of feeding, un-engorged mosquitoes were removed and engorged mosquitoes were maintained in the insectary under standard conditions [37] with daily access to a 10% sucrose solution or water and raisins. Midguts were dissected 9 days after the infectious bloodmeal, stained with 0.1% mercurochrome and the number of oocysts in each preparation counted. Uninfected bloodmeals were provided to transgenic and wild-type control mosquitoes following the membrane feeding. For the m4B7 25.1 experiments, mosquitoes were allowed to feed on the first and second days post-infection. Mosquitoes in the m2A10 experiments were allowed to feed on the 4^th^, 8^th^, and 12^th^ days post-infection. Engorged and un-engorged females were not separated after the uninfected bloodmeals in m2A10 experiments 1–4, while un-engorged females were discarded in experiments 5–7. Samples of wild-type control and m2A10 females were dissected for oocyst counts on the 10^th^ day post-infection. The salivary glands of all remaining m2A10 and wild-type control females were dissected 17–19 days post-infection. A hemacytometer was used in m2A10 experiments 1–4 to count salivary gland sporozoites [13]. The samples in experiments 5–7 were dried on 6 mm well slides and stored at −20°C. Sporozoites were stained using SlowFade Gold antifade reagent with DAPI (Invitrogen) and counted with a Zeiss Axioskop using the Axiovision camera and software. Sporozoites were counted using one of three methods, depending on parasite density. Method 1: If the number of sporozoites in each of five fields was counted, and a total of 3 or more sporozoites was found, an average sporozoite/mm^2^ measurement was calculated. When a field contained greater than 50 parasites, Improvision Volocity software was used to count the number of sporozoite nuclei in the DAPI image (Measurement protocol: 1. Find 2D nuclei: separate touching nuclei with a separation guide of 0.4 µm, reject nuclei with an area of less than 0.2 µm^2^. 2. Exclude objects by size: exclude objects >10 µm^2^). Method 2: If the 3 sporozoite requirement of method 1 was not met, fields were examined until 3 sporozoites were counted, and an average sporozoite/mm^2^ measurement was calculated. Sporozoite/mm^2^ values were used to calculate the total number of sporozoites present in the 6 mm^2^ slide well area. Method 3: If a total of 3 sporozoites was not found in up to 25 fields, the entire 6 mm^2^ slide well area was examined for an exact count. GraphPad Prism software was used to calculate statistical significance using Mann-Whitney *U* tests.

### Accession numbers

The GenBank (http://www.ncbi.nlm.nih.gov/Genbank/) accession numbers for the m1C3, m4B7, and m2A10 genes are HQ315886, HQ315885, and HQ315884, respectively.

## Supporting Information

Figure S1 Immunoblot loading controls. Coomassie-stained polyacrylamide gels used in immunoblot analyses of m2A10 expression. (A) Polyacrylamide gels used in m2A10 immunoblot presented in Figure 4A. (B) Polyacrylamide gel used in m2A10 immunoblot presented in Figure 4B. (C) A Coomassie-stained polyacrylamide gel loaded with an equal volume of each sample used in the immunblot of non-denatured hemolymph samples (Figure 3C) displayed protein migration. All labeling as in Figure 4.
(TIF)

Table S1 Comparison of *Anopheles gambiae* (256,340 codons) and *Mus musculus* (18,786,705 codons) codon usage for select amino acids [23].
(DOC)

Table S2 Transgene-specific primers used in expression analyses.
(DOC)
