# Supplementary figures and images for: Engineered Resistance to Plasmodium falciparum Development in Transgenic Anopheles stephensi
Source: PLoS Pathog. 2011 Apr 21;7(4):e1002017. doi: 10.1371/journal.ppat.1002017 (PMC3080844; doi:10.1371/journal.ppat.1002017)

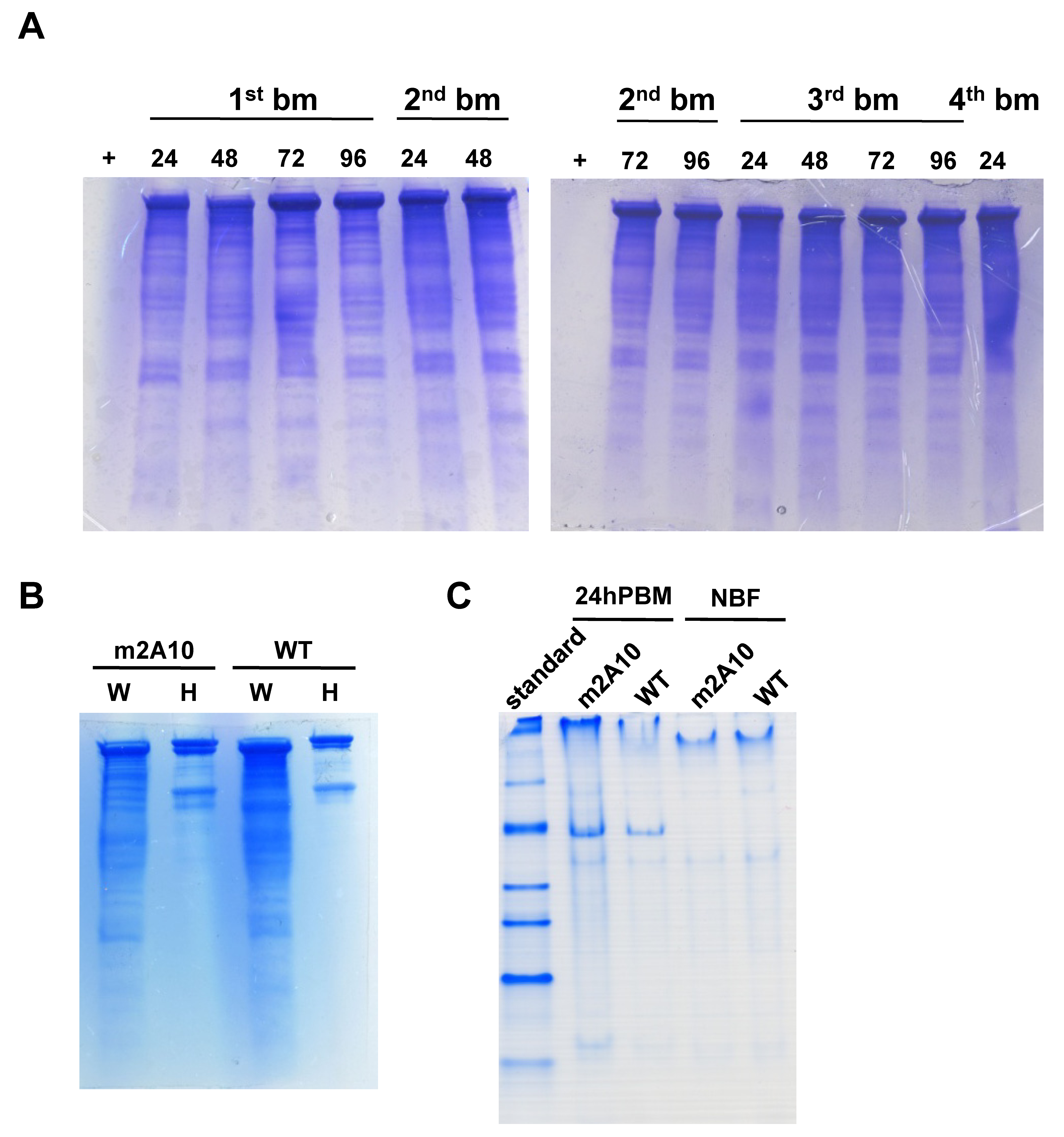

Supplement: Figure S1 — Immunoblot loading controls. Coomassie-stained polyacrylamide gels used in immunoblot analyses of m2A10 expression. (A) Polyacrylamide gels used in m2A10 immunoblot presented in Figure 4A. (B) Polyacrylamide gel used in m2A10 immunoblot presented in Figure 4B. (C) A Coomassie-stained polyacrylamide gel loaded with an equal volume of each sample used in the immunblot of non-denatured hemolymph samples (Figure 3C) displayed protein migration. All labeling as in Figure 4. (TIF) [file ppat.1002017.s001.tif]
