# Supplementary material for: Engineered Resistance to Plasmodium falciparum Development in Transgenic Anopheles stephensi
Source: PLoS Pathog. 2011 Apr 21;7(4):e1002017. doi: 10.1371/journal.ppat.1002017 (PMC3080844; doi:10.1371/journal.ppat.1002017)
Supplement: Table S1 — Comparison of Anopheles gambiae (256,340 codons) and Mus musculus (18,786,705 codons) codon usage for select amino acids [23]. (DOC) [file ppat.1002017.s002.doc]

**Table S1.** Comparison of *Anopheles gambiae* (256,340 codons) and *Mus musculus* (18,786,705 codons) codon usage for select amino acids [23]

| **Amino Acid** | **Codon** | **Species** | **Frequency/1000** |
| --- | --- | --- | --- |
| S | UCU | *M. musculus* | 16.1 |
|  | UCU | *An. gambiae* | 4.1 |
|  | UCG | *M. musculus* | 4.3 |
|  | UCG | *An. gambiae* | 21.0 |
| P | CCU | *M. musculus* | 18.4 |
|  | CCU | *An. gambiae* | 4.8 |
|  | CCG | *M. musculus* | 6.2 |
|  | CCG | *An. gambiae* | 26.3 |
| T | ACG | *M. musculus* | 5.8 |
|  | ACG | *An. gambiae* | 24.6 |
| A | GCG | *M. musculus* | 6.6 |
|  | GCG | *An. gambiae* | 25.6 |
| R | AGA | *M. musculus* | 11.6 |
|  | AGA | *An. gambiae* | 4.0 |
|  | AGG | *M. musculus* | 11.9 |
|  | AGG | *An. gambiae* | 3.7 |
