# Supplementary material for: Engineered Resistance to Plasmodium falciparum Development in Transgenic Anopheles stephensi
Source: PLoS Pathog. 2011 Apr 21;7(4):e1002017. doi: 10.1371/journal.ppat.1002017 (PMC3080844; doi:10.1371/journal.ppat.1002017)
Supplement: Table S2 — Transgene-specific primers used in expression analyses. (DOC) [file ppat.1002017.s003.doc]

**Table S2. Transgene-specific primers used in expression analyses**

| **Primer name** | **Primer sequence** | **Annealing temperature (°C)** |
| --- | --- | --- |
| **RT-PCR** | | |
| m2A10 FOR | 5’-GAGACGGTGAAGATCTCGTGCAAGG-3’ | 67 |
| m2A10 REV | 5’-GCTTCGTACCACCACCGAACACGTA -3’ |  |
| m4B7 FOR | 5’-CAGCGATTACGGCATG-3’ | 56 |
| m4B7 REV | 5’-GCGCTGCAGACAGTAGTAA-3’ |  |
| m1C3 FOR | 5’-AAGGGCTCGCTGAAACTGT-3’ | 59 |
| m1C3 REV | 5’-ACAGAAGTACACCGCCAGGT-3’ |  |
| AsVg1 FOR | [22] | 67 |
| AsVg1 REV | [22] |  |
| AsCPA FOR | [31] | 59 |
| AsCPA REV | [31] |  |
| **RT-qPCR** | | |
| m1C3 FOR | 5’-AATCGAAGGGCTCGCTGAAACTGT-3’ | 60 |
| m1C3 REV | 5’-AGGTACAGCATGCTCTGCGAATCA-3’ |  |
